# Do platelets LINE up for aging?

**DOI:** 10.18632/aging.101636

**Published:** 2018-11-06

**Authors:** Hansjörg Schwertz, Matthew T. Rondina

**Affiliations:** 1 University of Utah Molecular Medicine Program, University of Utah, Salt Lake City, UT 84108, USA; 2 Rocky Mountain Center for Occupational and Environmental Health, University of Utah, Salt Lake City, UT 84108, USA; 3 Departments of Internal Medicine and Pathology, University of Utah, Salt Lake City, UT 84108, USA; 4 Department of Internal Medicine and GRECC at the George E. Wahlen Salt Lake City VAMC in Salt Lake City, UT 84148 USA

**Keywords:** platelets, aging, senescence, LINE

In early 2018, our group reported [1] that human and murine platelets contain functional endogenous reverse transcriptase (eRT), encoded by long interspersed nuclear element-1 (LINE-1/L1, a non-LTR retrotransposon implicated in the regulation of gene expression). We identified that in people living with HIV/AIDS (PLWHA) who were treated clinically with RT inhibitors, platelet activation and protein synthesis were upregulated compared to healthy donors without HIV. eRT activity in platelets orchestrated the formation of RNA-DNA hybrids, which served as a translational block. Treating platelets from HIV-negative donors with RT inhibitors *ex vivo* phenocopied our findings in PLWHA, including the upregulation of platelet activation responses and lifting of the RNA-DNA hybrid-induced translational block. We also found that inhibition of eRT *in vivo* accelerated mortality from platelet-dependent thrombosis. Together, this provided new evidence that platelets possess L1-encoded eRT activity regulating platelet activation and thrombosis. Furthermore, eRT in platelets also serves as a novel mechanism of translational control through the formation of RNA-DNA hybrids. Our findings also support published studies of platelets in HIV, where PLHWA have, in general, evidence of increased platelet activation, mitochondrial stress, and reactive oxygenation species generation [2,3].

Therapeutic advancements have transformed HIV from a terminal illness to a manageable chronic disease. However, PLWHA experience many features of accelerated aging [4], including systemic inflammation and atherothrombosis – even during sustained viral suppression. Furthermore, cardiovascular disease is currently the leading cause of death in PLWHA treated with ART [5]. The mechanisms driving these features of accelerated aging remain incompletely understood. Injurious platelet activation may contribute the increased risk of thrombo-inflammation in PLWHA. This seems to be especially relevant as established and emerging data highlight the central role of platelets in thrombotic, inflammatory, and immune processes, all pathways connected to cellular senescence.

Cellular senescence is implicated in disease development through a number of mechanisms, including the release of proteases and pro-inflammatory cytokines, with subsequent detrimental effects locally and systemically on the host. Cellular senescence was first described during *in vitro* experiments, where it could be triggered by excessive cellular passaging [4]. Since its discovery, several types of cellular senescence have been described, including oncogene-induced senescence (OIS) and stress-induced premature senescence (SIPS) [4]. Of note, SIPS is induced in response to oxidative stress, altered proteasome function, and medication toxicities. Senescence has also been linked recently to vascular aging and CVD [6].

Notably, several drugs used as part of ART treatment are linked to senescence. For example, nucleoside reverse transcriptase inhibitors (NRTIs) induce senescence in a mammalian target of rapamycin complex 1 (mTORC1) dependent fashion [7]. This mechanism can be mediated by interference with translational control pathways as well as deregulated autophagy, a process involving the orderly degradation of cellular components. Autophagy can be an adaptive response to stress, although in some settings it may promote cell death. In this regards, it is intriguing that one of the proteins we identified as translationally regulated by LINE-1 induced RNA-DNA hybrids was MAP1LC3B (microtubule associated protein 1 light chain 3 beta, also referred to as LC3). LC3 is a central protein in the autophagy pathway [1].

As PLWHA are often taking a combination of anti-viral drugs, it is difficult to disentangle the effects of single agents on cellular senescence *in vivo*. Moreover, the mechanisms behind the accelerated aging complications in PLWHA, including cardiovascular disease, is complex and multifactorial. While still incompletely understood, this increased disease burden may involve some traditional CVD risk factors, as well as emerging factors associated with HIV itself, such as chronic immune activation, inflammation, and metabolic dysregulation.

In summary, platelets are abundant, anucleate cells with a dynamic repertoire of functions that span thrombotic, inflammatory, and immune pathways. Platelets secrete pleiotropic immune and inflammatory mediators that orchestrate heterotypic interactions with endothelial cells, monocytes, neutrophils, and other cells [8]. While still incompletely understood, platelet activation in PLWHA on ART may contribute to premature senescence and an increased cardiovascular risk [2]. Our report [1] adds another facet to this intriguing interplay, demonstrating that platelets possess eRT that functionally serves to regulate platelet activation and protein synthesis. Inhibition of eRT, as occurs in PLWHA on ART, led to increases in the synthesis of the autophagy protein LC3, as well as platelet activation and thrombosis. This newly deciphered mechanism underscores the versatility of platelets and raises new questions on how platelets may influence aging and senescence.
